# The role of expectations, hype and ethics in neuroimaging and neuromodulation futures

**DOI:** 10.3389/fnsys.2014.00214

**Published:** 2014-10-31

**Authors:** Elena Rusconi, Timothy Mitchener-Nissen

**Affiliations:** ^1^Department of Security and Crime Science, University College LondonLondon, UK; ^2^Division of Psychology, Abertay UniversityDundee, UK; ^3^Department of Neurosciences, University of ParmaParma, Italy; ^4^Department of Science and Technology Studies, University College LondonLondon, UK

**Keywords:** expectations, hype, ethics, brain modulation, neuroimaging, deception

## Abstract

The production of *expectations* or future-goals for the development of techniques which “read” and modulate brain function, represent an important practical tool for neuroscientists. These visions-of-the-future assist scientists by providing focus for both individual and cross-disciplinary research programs; they encourage the development of new industrial sectors, are used to justify the allocation of government resources and funding, and via the media can help capture the imagination and support of the public. However, such expectations need to be tempered by reality. Over-hyping brain imaging and modulation will lead to disappointment; disappointment that in turn can undermine its potential. Similarly, if neuroscientists focus their attention narrowly on *the science* without concomitant consideration of its future ethical, legal and social implications, then their expectations may remain unrealized. To develop these arguments herein we introduce the theoretical concept of expectations and the practical consequences of expectations. We contextualize these reflections by referring to brain imaging and modulation studies on deception, which encompass the measurement-suppression-augmentation range.

## Introducing expectations

On May 25th in 1961 during a special address to Congress, a young and charismatic US President J.F. Kennedy presented an incredibly exciting prospect to Congress and the entire nation: a manned moon landing. He was asking Congress to share his vision, and in so doing to invest an astronomical budget in research and development to increase the likelihood of that vision becoming reality. Less than a decade later that vision was achieved with the Apollo 11 mission and the first moon walk: “one small step for man, one giant leap for mankind” (Neil Armstrong, 1969). Kennedy was perfectly aware that the required technology was not yet available in 1961 but he was well informed about its potential and knew a thing or two about the power of expectations.

Proclaiming the start of the Decade of the Brain on 1st January 1990, President Bush senior stated “The cooperation between […] agencies and the multidisciplinary efforts of thousands of scientists and health care professionals provide powerful evidence of our nation’s determination to conquer brain disease”. He raised public awareness and brought to the fore a series of targets (e.g., stopping or curing Alzheimer’s disease), which have not been quite met yet but the attention and investments brought upon relevant neuroscientific research see us at a clear advantage compared to our colleagues trying to meet the same targets 25 years ago.

When discussing scientific and technological advancement the concept of *expectations* adopts the shape of; goals, promises, visions of a future reality, “wishful enactments of desired futures” (Tutton, 2011, p. 413), and “real-time representations of future technological situations and capabilities” (Borup et al., 2006, p. 286). The value of such expectations, as a powerful precursor to future scientific and technological advances, cannot be underestimated as both a conceptual and a practical tool.

In this paper we begin by setting out the benefits to neuroscientists of employing *expectations*. This positive message is then balanced by a discussion of *hype* and its potential negative connotations for research programs. We finish by positing that if neuroscientists temper their expectations of future scientific and technological advances with social, ethical, and legal concerns, they will be better placed to avoid the drawbacks associated with hype while maintaining their ability to enjoy the benefits expectations can bring.

## Expectations: conceptual and practical benefits

On a conceptual level, expectations (as described above) represent *the potential pre-manifestation* or *genesis* of a future scientific or technical advance which has yet to be attained. To illustrate; neuroscientists envision a future whereby neuroimaging and brain stimulation techniques have been developed which, say, detect deception and/or increase the disposition and the ability to lie and deceive (Karim et al., 2010; Mameli et al., 2010; Karton and Bachmann, 2011), “simply and safely influenc[e] the human will and freedom by interfering with deception” (Priori et al., 2008, p. 455; see also the “deception inhibitor”: Bohning et al., 2004). They may also envision military applications whereby the ability of human operators to sense and assess threats in the real world is enhanced by noninvasive brain stimulation (e.g., Parasuraman and Galster, 2013). These particular near-to-medium term expectations are typically based on converging evidence from a small series of original precursor studies on human participants conducted in one or more laboratories around the world. When neuroscientists envision a future whereby in-helmet ultrasound transducers could be used to remotely control brain activity (Tyler, 2010; Sato et al., 2014), or where a time-slowing pill may be used in law enforcement and prisoner incarceration (Heaven, 2012; Anderson, 2014), or “brain prostheses” can be used by the military to enhance memory for complex environments or existing memories can be wiped out from the brain of personnel under capture (Moreno, 2012) they are envisioning medium-to-long term expectations. Here the know-how needed to achieve these particular goals remains largely unavailable and it is claimed that the ethical and legal implications of such enterprise are still relatively imponderable (Attiah and Farah, 2014).

What makes these particular expectations of specific interest to neuroscientists is that through a combination of their training, expertise, accumulated knowledge, and access to funding and specific equipment, neuroscientists find themselves well placed to successfully act and bring these expectations and visions into reality. This is not to claim that they are the *only scientists* capable of fulfilling these expectations; merely that they may enjoy a head start on their competitors when attempting to do so.

Moving beyond the conceptual, it is via its practical benefits that the true power of expectations becomes recognizable; for expectations possess the power to assist turning future-visions into tangible advancements. With similarities to the concept of the *self-fulfilling prophecy* (see Merton, 1948), future expectations have been described as possessing performative or generative powers (Borup et al., 2006) in that they are “crucial to providing the dynamism and momentum upon which so many ventures in science and technology depend” (Brown and Michael, 2003, p. 3). By studying past advancements, it has been recognized that expectations can provide the following benefits (see: van Lente, 1993; Hedgecoe and Martin, 2003; Borup et al., 2006; Konrad, 2006; Pollock and Williams, 2010; Bakker et al., 2012):

- A focal point to guide and drive research activities, as well as a common vision/bridge with which to enhance inter-disciplinary interactions and communication.
- A promise of future benefits with which to justify and legitimize the mobilization and allocation of financial resources and other forms of support.
- The means of attracting the attention and engagement of diverse actors and potential collaborators; from scientists to investors, governments, NGOs and policy actors.
- They enable the production of experiments, models, research projects, and calculations.
- The construction of future scenarios for shaping a technology, as well as the formation of a consensus or structure to counterbalance the inherent uncertainty over the potential of a new technology which is highest during the early development stages.
- A means of enlisting public support through the presentation of possible future(s).

Within the life sciences expectations of future medical advancements have repeatedly been employed by specific research areas seeking to benefit from the points listed above. These expectations have included; the promise of lives being saved and the development of new cures for diseases as the result of human embryonic stem cell research (Rubin, 2008); the development of anti-aging treatments which will extend human lives (Mykytyn, 2010); and the expectation that by identifying one’s genetic makeup, pharmacogenetics will deliver bespoke, personalized medications (Limdi and Veenstra, 2010).

Within neuroscience, and in particular the study of neuroimaging and brain stimulation, we can readily identify how neuroscientists are employing expectations in their efforts to secure supporters and resources for research, attract collaborators and potential customers for spin-off businesses, and provide focal points with which to guide research. All of this is achieved through the presentation of possible futures where brain imaging and modulation advances have been successfully developed. What follows is a brief description of some of these research programs, accompanied by the envisioned advances (the expectations) they are predicted to produce. While presenting these, we will intentionally embrace such expectations (whether or not we believe they are realistic), as this will help making the points we are raising in the following sections even more obvious.

## Expectations about the future of brain imaging and modulation: the case of deception

Riding a wave of exciting technical developments in neuroimaging, one popular research enterprise that has provided fertile ground for boosting expectations on a global scale is the study of deception. Here deception is defined as the concealment of the truth, which often takes the form of a memory, in order to mislead. On average humans are thought to be badly equipped to detect deception when the person attempting to deceive is unfamiliar to the observer (Bond and DePaulo, 2006). Expectations in this area have included a promise of a future whereby the scientific detection of deceptive claims and behaviors would add a game-changing weapon to the arsenal of forensic sciences, police, the judiciary, and military and security agencies worldwide. Whether that can be ever achieved is still open to speculation.

Historically a multitude of deception detection systems have been invented and employed by public and private bodies with variable success. Few such systems survived in the present; and none has been scientifically proven to solve the deception detection problem in the real world. The most popular system so far has arguably been the polygraph, however, on close inspection, it was deemed unreliable; though since its myth is very hard to debunk, it still has use as a deterrent (NRC (National Research Council), 2003; see also Moreno, 2012). More recently, advances in functional Magnetic Resonance Imaging (fMRI) and neural decoding techniques prompted expectations that mind-reading (or more accurately brain-reading; Gazzaniga, 2005) may soon become reality (Haynes and Rees, 2006). In relation to deception, expectations and research have focused on systems that can accurately detect deceit from brain activation contrasts or pattern analysis, perhaps in connection with other physiological measures (Kozel et al., 2009; Langleben and Moriarty, 2013). These would help reduce uncertainty and provide information otherwise undetectable to the interviewer. By making the interviewee’s brain *more accessible* they would artificially augment the interviewer’s detection capabilities. Moreover, they would provide essential information about the localization of deception-relevant brain activity for individually targeted deception modulation tools (see e.g., Bohning et al., 2004). It may be easy to create expectations about potential advantages for the criminal justice system and security at large if such an enterprise were to actually succeed. For example, it could be claimed that it will be possible to accurately discriminate between genuine and concocted eyewitness testimony (Azar, 2011), more judiciary decisions could be based on objective evidence, and trials would become more expedite. Culprits would be less likely to elude unmasking and arguably more likely to be found guilty; crime rates may drop; and organized crime which relies on member loyalty could be more penetrated.^1^ On its face, and without critical analysis, the benefits cited here might be presented as both obvious and great in number.

Expectations within deception research are by no means limited to the development of reliable lie detectors. Indeed noninvasive brain stimulation (e.g., tDCS and TMS) may soon offer both a new, upgraded alternative to truth serums by enabling the user to interfere with activity in brain regions that are causally related with deception (e.g., Priori et al., 2008; Karton and Bachmann, 2011), as well as its counterpart; a portable deceit enhancer (Karim et al., 2010; Mameli et al., 2010; Karton and Bachmann, 2011; Fecteau et al., 2013). It is easy to imagine the enormous advantages for counterespionage, policing, and diplomacy if a “deception inhibitor” was available. In extreme scenarios, interrogations would become more humane; negating the need for more outrageous practices such as sleep-deprivation, electric shocks, nail pulling, water-boarding, and so forth, to weaken psychophysical defense mechanisms and coerce the disclosure of information (see e.g., Tarabay, 2014, and the documentary Standard Operating Procedure, 2008). In more ordinary cases, this induced cooperation would make criminal justice mechanisms more agile and cost-effective. Potentially more guilty individuals will confess and be rightfully convicted.

Similarly, it is arguable that difficult diplomatic negotiations could benefit from both sides employing “deception inhibitors”. These may decrease the capability of either side negotiating in bad-faith by concealing their true thoughts (though it is equally likely such devices may make parties reluctant to enter into negotiations to begin with). Conversely “deception boosters” would conceivably be invaluable in the hands of private and governmental agencies concerned with security, policing, espionage, and diplomacy. Undercover agents might be better able to conceal their true identities, and negotiators better equipped to convincingly promote positions they knew to be false.

## Hype: the downside of expectations

**Figure 1:**
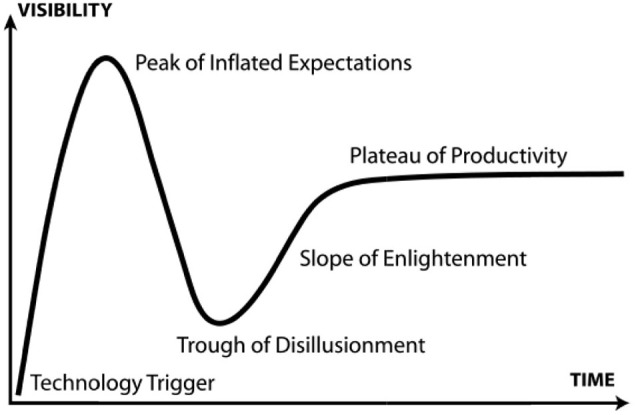
**The Gartner Hype Cycle (van Lente et al., 2013)**.

While expectations such as these play important roles in achieving scientific and technological advancements, we must acknowledge an inherent fundamental limitation: expectations may be unreliable as predictors of the future (Brown and Michael, 2003). Even when high expectations for future advancements are shared by a large multidisciplinary community of scientists enjoying both support and resources, this does not guarantee scientific success (Pollock and Williams, 2010; Tutton, 2011). The failure of a scientific or engineering endeavor to meet its expectations leads to accusations of *hype*. Used in this way hype is referring to unrealistic, unattainable expectations; hence it is often only a distinction in framing that separates expectations from hype. What follows the characterization of (positive) expectations as (negative) hype is usually a period of disappointment or disillusionment. Enthusiasm for the proposed scientific advancement plummets as a result of expectation not being met, possibly followed by a gradual form of recovery. This process is referred to as a hype-disappointment cycle (van Lente et al., 2013). The Gartner hype cycle (see Figure 1), employed by the analyst firm Gartner Inc. is probably the most recognized model employing this concept. Note that under this model, the failure of scientific research or technological development to meet its initial expectations does not mean it is automatically and permanently discarded; rather that it is subject to a period of slower development towards more realistic goals.

This process, whereby (what in hindsight proved to be exaggerated) expectations were employed to secure support for research only to be followed by an abrupt collapse, has repeated itself in a multitude of past technologies; including hydrogen-powered cars (Bakker, 2010), nanotechnologies (Ebeling, 2008), and genomics (Bubela, 2006). No scientific discipline is immune from such outcomes, and some expectations for the promise of brain imaging and modulation will almost inevitably suffer the same result to some extent (see e.g., Walsh, 2013). The likelihood of this occurring is growing as neuroscientists increasingly push the boundaries of what they are presenting as realistic research outcomes in their efforts to secure support and resources for their research. Indeed in Gartner’s (2013) Hype Cycle for Emerging Technologies, *human augmentation* (which would include brain augmentation) is specifically identified as a technology currently *on-the-rise* towards its peak of inflated expectations (Gartner, 2013). It has also been suggested that *functional neuroimaging* technologies may be even further advanced along the classic Hype Cycle trajectory than augmentation (Rachul and Zarzeczny, 2012). One example from neuroscience justifying this characterization is the potential use of fMRI in the detection of deception.

More than a decade on since the first peer-reviewed empirical reports on deception detection in fMRI (see e.g., Spence et al., 2001; Langleben et al., 2002; Ganis et al., 2003), the field has seen incremental advances both in the “brain-reading” technique and in the refinement of cognitive testing protocols (see e.g., Sartori et al., 2008; Moreno, 2012; Wright et al., 2012; Park and Friston, 2013). Multiple patent applications have been filed but the envisioned fMRI deception detector looks still far from becoming reality. We have highlighted elsewhere a series of hurdles that an fMRI-based deception detector would need to surmount before it can succeed (Rusconi and Mitchener-Nissen, 2013). These include scientific hurdles such as; the assumptions and inferences underlying fMRI processes, the need to achieve consistent internal validity, manipulation of results by subjects, and the difficulty in moving beyond the laboratory setting. Also included were legal and ethical hurdles, such as; possible human rights violations, the issue of compelled questioning, the probative value of such evidence, and how the right to a fair trial would be impacted. It is open for debate whether or not its stimulation counterpart stands a better chance to translate into an applied deception modulator tool. One obvious advantage is that certain stimulators are both portable and affordable; however the science is probably not mature enough to justify its near-medium term expectations. The reported modulatory effects (facilitation or interference) obtained in controlled laboratory settings are statistically significant but rather minuscule in a standard interrogation context and short-lived, thus as things stand the gains may not eventually justify the efforts. This consideration only applies to applicative expectations—not to the utility and importance of such research within basic science. The in-lab testing protocols have not been validated against real-world situations (Luber et al., 2009), and the relation between modulation of deceits and its effects on other brain functions are still unclear, as is the role of individual differences (see Levasseur-Moreau et al., 2013 for a thorough discussion).

## The downside of hype

The reason why neuroscientists should resist the temptation to over-hype the potential for brain augmentation is the detrimental consequences that can follow once unrealistic expectations are inevitably unmet. These include the following (see Brown, 2003; Ruef and Markard, 2010):

- Unrealistic short-term hype detracts from the long-term value of the basic science.
- Financial losses for corporate investors, and future difficulty in securing resources within fields which have suffered from a collapse of expectations.
- Resources are diverted from more realistically-achievable, yet less exciting, research programs.
- Undermined trust, which can slow or prevent future progress within a field of research.
- The reputations of individuals, companies/institutions, and entire research fields can suffer as past promises fail to materialize in the present.
- Identifiable people can suffer as a result; from individuals who suffer financial loss from investing in unrealistically over-hyped research programs, to patients who suffer emotionally having believed the claims that certain proposed brain augmentation techniques could treat their conditions.

In different combinations, these detrimental consequences arise in the multitude of science and technology examples presented in the annual Gartner hype cycles, as well as the specific case-studies of hydrogen-powered cars, genomics, and nanotechnology discussed above.

Nevertheless, despite all these drawbacks of hype the temptation still remains to present the most ambitious expectations for scientific and technological research as something achievable in the future; be they time-slowing pills to replace prison sentences, portable reliable lie detectors, brain control devices, or extra-memory microchips. Perhaps unsurprisingly the probability of achieving these goals (which may be incredibly small) and the time-frame for doing so (which may be incredibly long) are afforded little discussion. The challenge facing neuroscientists is how to construct and present expectations that are less likely to cross that invisible barrier dividing “hope” from “hype”.

## Addressing ethical concerns to produce better expectations and the risks of not doing so

It is our contention that by reflexively considering the ethical, legal, and social impacts of a potential future technology or field of advancement, neuroscientists can improve the quality of the expectations they disseminate within their own field; thereby developing more nuanced, better expectations. This of course does not replace or neglects but is additional to the need for neuroscientists to educate the media, patients and research subjects about the potential and limits of neuroscience and neuroscientific techniques; to conduct enough studies and generate valid scientific data to know what neuroscience and these techniques can and cannot do. Before we develop this contention below, two points require expansion and clarification here. Firstly, we are characterizing these expectations as “better” if they constitute visions of the future constructed after taking into account existing legal regimes and concepts of human rights, ethical norms, and attitudes of societies into which they are expected to operate. Secondly, it should also be emphasized that the stage we are referring to is the formulation and presentation of initial expectations by the scientists. This stage often precedes wider public/multi-stakeholder engagement and the development of respective communication strategies (for more detailed discussions of such topics see: Jasanoff, 2003; O’Doherty and Einsiedel, 2012; Bucchi and Trench, 2014; Stilgoe et al., 2014).

The concomitant social, legal and ethical implications of a proposed scientific/technological expectation are often neglected at the initial stage of its formulation and proposition. Hence what is often initially presented is the envisioned advancement free from ostensibly non-scientific constraints; i.e., presenting the expectations of a pill that slows one’s perception of time or of the portable lie detector device, but doing so free from any contextual discussion. This may occur for a number of reasons, including: (a) because no ethical/legal/social implications exist (highly unlikely for most expectations); (b) those presenting these futures have not considered and/or foreseen such implications; (c) these implications are viewed as either unimportant or not the responsibility of the scientist involved; and/or (d) there is a fear that by acknowledging the existence of such implications, the advancement in question will be viewed less positively thus putting at risk the benefits expectations can bring to a research program.

However, failing to reflexively consider the ethical, legal, and social impacts of a potential future technology or field of advancement does not negate their existence or make them disappear. These factors are going to have to be addressed at some point in the research and development lifecycle of a new technology for scientific advancements do not develop or exist separately to the societies in which they operate (see MacKenzie and Wajcman, 1999). We contend that both the benefits of engaging in this reflexive process early in the development of new expectations, as well as the potential negative implications of failing to do so, make this early consideration of ethical, legal, and social impacts an essential step in their development.

By considering ethical, legal, and social impacts to create “better” expectations, neuroscientists gain a number of advantages. They can act to avoid their expectations becoming characterized as hype. They can identify sources of possible controversy and resistance within their proposed expectations, thereby affording them the opportunity to consciously address such issues both through how they formulate their expectations and how they seek to present them. Also by mitigating the propensity for individuals or societal groups to actively criticize or resist their expectations, the neuroscientist is effectively maximizing their potential network of future supporters.

Failure to create “better” expectations can have specific negative consequences. It can shift expectations into the realm of hype (the inherent negative consequences of such were discussed earlier). It has also been posited that by presenting technological expectations unfettered by non-technical considerations (such as ethics, legality, and social acceptability), critics can more easily create counterarguments by presenting dystopian futures involving them (see Tutton, 2011). Essentially the argument here is that; if the presentation of an expectation devoid of some inherent moral character justifies utopian visions of its future use, then dystopian visions of its future use are also equally valid (see Pickersgill, 2011 for a practical example of this argument in relation to *neuro-law*).

Furthermore, by promoting a real-world version of, and uses for, a future technology absent of a robust consideration of social, legal and ethical issues is to promote a tool that is more easily open to legitimate social, legal and ethical criticism. We believe this advice has resonance for many of the expectations currently being espoused by neuroscientists within the field of neuroimaging and brain modulation; from enhancing the cognitive potential of users, to brain control/manipulation, to increasing/decreasing the capacity of an individual to deceive. An important point here is that each expectation must be individually reflexively examined, for the nature of these social, legal and ethical criticisms will differ depending on the specific application that is being envisaged. This diversity means there are no shortcuts to robust reflexive examinations. For example, in the case of fMRI deception detection in criminal proceedings discussed above, we examined in depth how future expectations of this advancement conflicted with existing legal and human rights regimes, as well as social and ethical norms. This led us to conclude that “[c]ognitive neuroscientists must be careful not to over-play what these technologies can offer criminal courts nor their vision of the potential future role of neuroscience within criminal courts, lest they overplay themselves out of the courtroom altogether” (Rusconi and Mitchener-Nissen, 2013, p. 9). Conversely, the main hurdles of brain reading and modulation may be characterized as: legal and human rights issues over compelled questioning, fair trials, and the probative weight attached to such evidence; as well as the social implications of undermining our naturally evolved capacity to deceive. Whereas those raised by cosmetic enhancement include: the ethicality of altering fundamental elements of self-identity; social justice concerns over the inequitable distribution of cognitive enhancement techniques; the undermining of autonomy; and determining the “proper” role of enhancement technologies in society (Wolpe, 2002; Hamilton et al., 2011). Given the mounting emphasis on “impact”, even from government founding agencies that have traditionally encouraged basic research, neuroscientists may need some assistance in this reflexive process. This could be offered in the form of further training and multidisciplinary education opportunities or direct access to professional figures with the relevant expertise. On the other hand, funding agencies and scientific journals could also be required to closely monitor and develop awareness of the more hype-prone research areas, as their endorsement (sometimes motivated by the immediate visibility that inflated expectations can grant) may play a role in amplifying, rewarding and maintaining hype within the scientific community.

## Conclusion

Expectations are presented as a valuable tool for neuroscientists in acquiring both resources and networks of allies to assist in converting these expectations into reality. However, in applying the often-used adage of caution, that “if a little bit of something is good then a lot is not necessarily better”, unrealistic expectations can become synonymous with hype which in turn can threaten the very programs the scientists are seeking to promote. Similarly, promoting the expectation of a scientific or technological advancement free from any social, legal or ethical considerations will not benefit that advancement. For any critical assessment will easily be able to identify these non-technical concerns which neuroscientists have ostensibly ignored; again placing at risk the programs they are seeking to advance.

Provided they are supported by (and are not used as a replacement for) sound research on the safety and efficacy of neuroscientific techniques, expectations *are* a valuable resource for capturing the public’s imagination and promoting investment in an area for research. We encourage those neuroscientists working in the field of neuroimaging and brain modulation, with its exciting prospects for the future, to employ this valuable resource as they would any other at their disposal to help further their research goals. We simply caution that when doing so, if they present expectations that are disassociated from any measure of realism, or treat the concomitant legal, social and ethical considerations as optional extras, then hype (and Gartner’s *Trough of Disillusionment*) surely awaits.

## Conflict of interest statement

The authors declare that the research was conducted in the absence of any commercial or financial relationships that could be construed as a potential conflict of interest.

## Footnotes

^1^See http://www.fbi.gov/about-us/investigate/organizedcrime
